# Supplementary material for: Thermochromic hydrogel with high transmittance modulation and fast response for flexible smart windows
Source: Commun Mater. 2025 Nov 4;6(1):239. doi: 10.1038/s43246-025-00956-3 (PMC12586165; doi:10.1038/s43246-025-00956-3)
Supplement: Supplementary file 1 — Supplementary Information [file 43246_2025_956_MOESM1_ESM.pdf]

Supplementary Information

Thermochromic Hydrogel with High Transmittance Modulation and Fast Response for Flexible Smart Windows

Fan Jiang[1^], Kui Yu[1*^], Roland Kieffer[1], Djanick de Jong[1], Richard M. Parker[2], Silvia Vignolini[2,3], Marie-Eve Aubin-Tam[1*]

[1]Department of Bionanoscience, Kavli institute of Nanoscience, Delft University of Technology, Van der Maasweg 9, 2629 HZ, Delft, The Netherlands

[2]Yusuf Hamied Department of Chemistry, University of Cambridge, Lensfield Road, Cambridge, CB2 1EW, United Kingdom

[3]Sustainable and Bio-inspired Materials, Max Planck Institute of Colloids and Interfaces, Potsdam 14476, Germany

*k.yu-2@tudelft.nl (K. Yu), m.e.aubin-tam@tudelft.nl (M. E. Aubin-Tam)

^these authors contribute equally

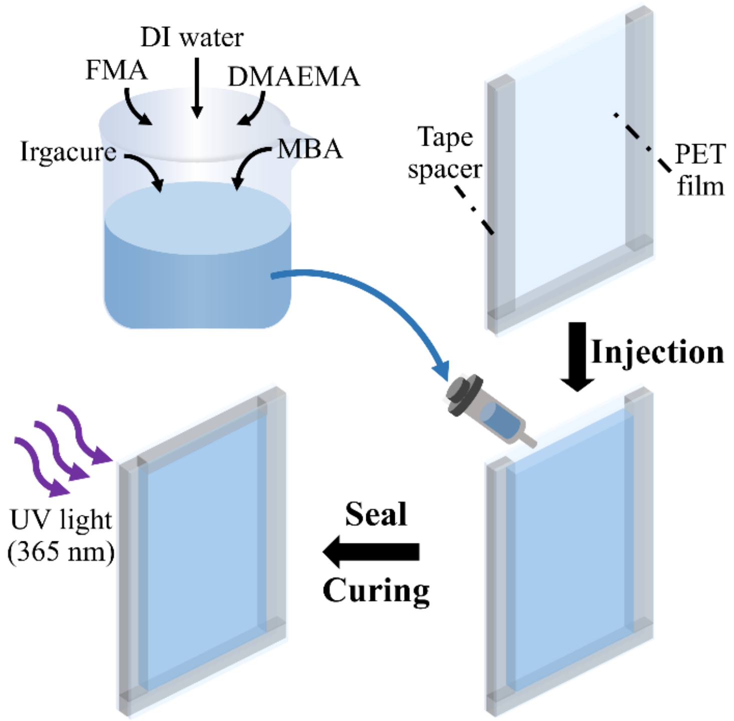

Supplementary Fig. 1: Schematic illustration of the preparation process for the DMFM hydrogel device.

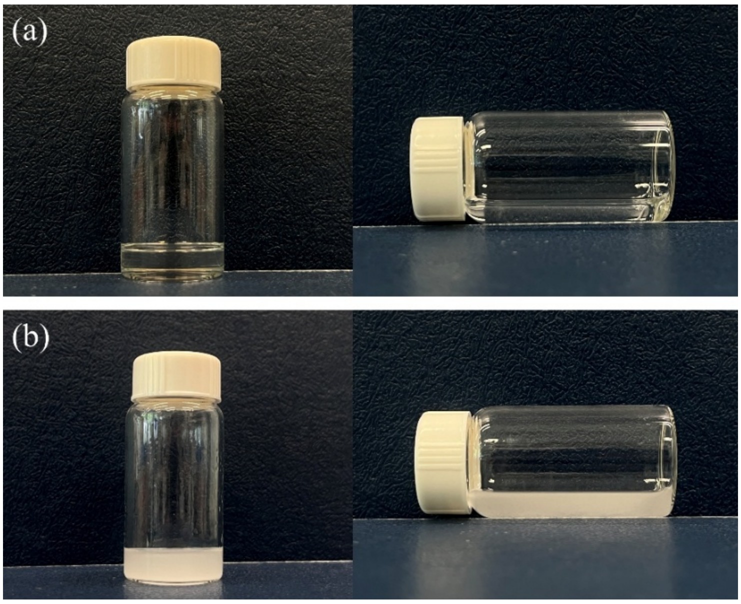

Supplementary Fig. 2: Uncured DMFM-4 precursor solution. (a) Photographs of uncured DMFM-4 precursor solution at transparent state at T= 20 °C; and (b) at turbid state at T= 30 °C in a 20 mL glass vial in standing positions (left) and in horizontal positions (right).

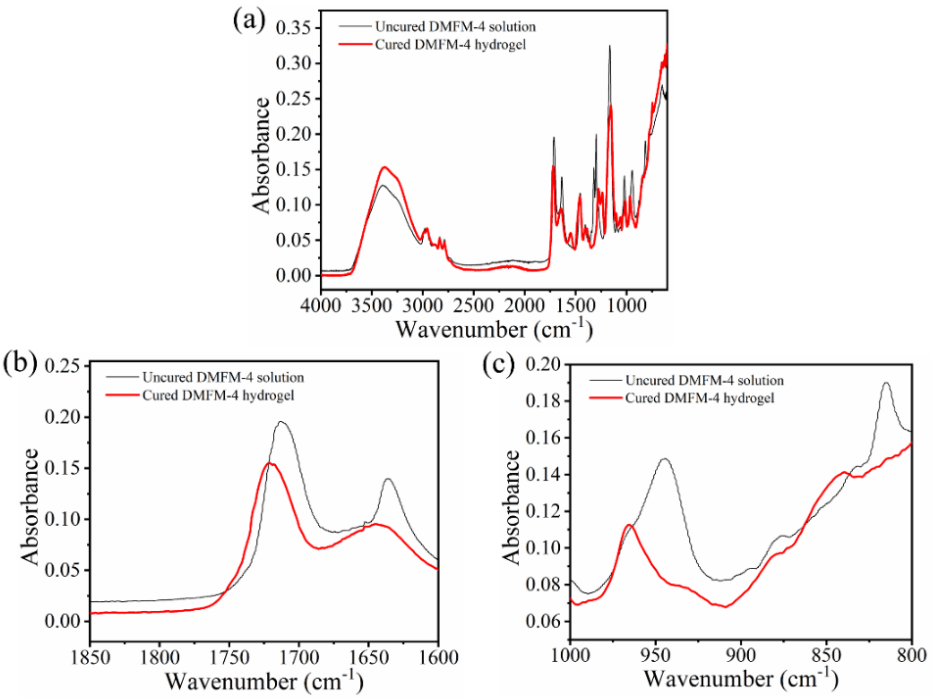

Supplementary Fig. 3: FT-IR spectra of DMFM-4 precursor solution (grey) and corresponding cured hydrogel (red). Key spectral regions are highlighted: (a) 4000–600 cm^{-1}; (b) 1850–1600 cm^{-1}; (c) 1000–800 cm^{-1}.

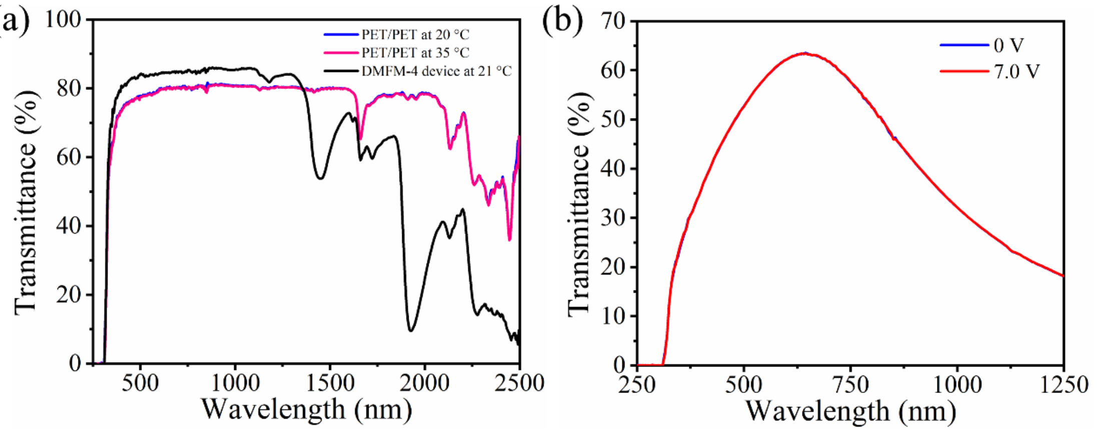

Supplementary Fig. 4: Optical properties of PET and ITO/PET films. (a) Solar transmittance spectra (250 to 2500 nm) of an empty PET/PET frame with a spacer thickness of 0.5 mm recorded at 20 °C (blue) and 35 °C (red), The lower transmittance in the 300-1300 nm region compared to the DMFM-4 device at 21 °C (black) is likely due to light scattering at the air-PET interfaces. (b) Optical transmittance of the empty ITO-PET/PET frame with a spacer thickness of 0.5 mm when either no voltage (blue) or a voltage of 7.0 V (red) was applied.

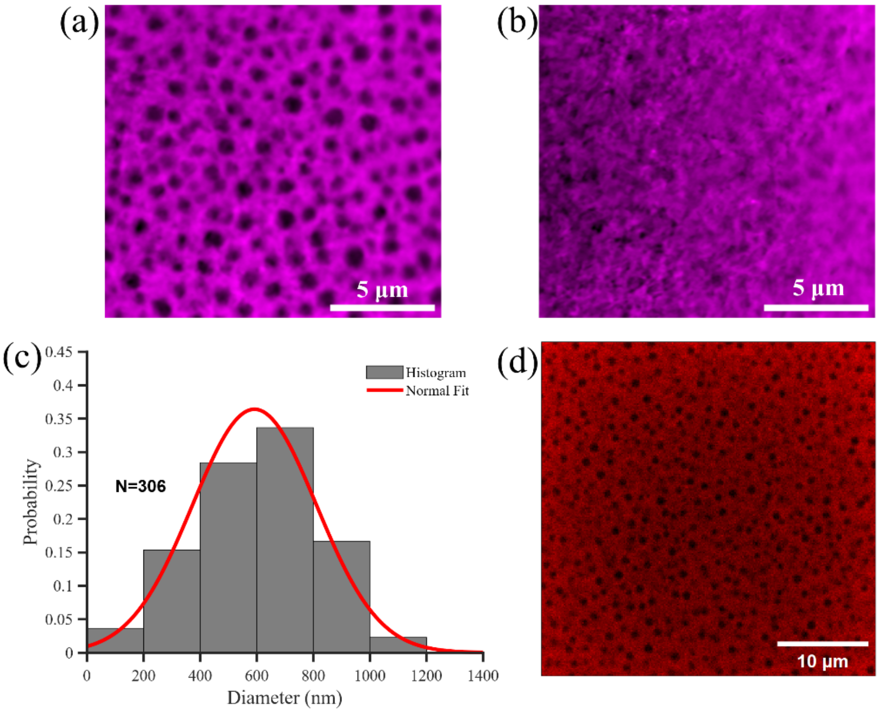

Supplementary Fig. 5: Confocal microscope images and particle distribution analysis of DMFM-4 hydrogel. (a) Confocal microscope images of DMFM-4 hydrogel device with Rhodamine B (hydrogel thickness: 0.5 mm) above transition temperature, at T= ~50 °C; and (b) below transition temperature, at T= 20 °C. (c) Size analysis of dark regions in the DMFM-4 hydrogel devices, at T= ~50 °C (N = 3 DMFM-4 hydrogel devices, data represent the combined results of 3 samples). Using ImageJ, the diameters of the cross-section of these water cavities were calculated, assuming perfectly spherical particles. The imaged cross-sections have diameters mainly ranging from 400 to 800 nm which accounted for 62.1% of statistical distribution, and cross-sections with diameters from 800 to 1200 nm constituted 19.0%. (d) Confocal microscope images of DMFM-4 hydrogel device with Nile Red (hydrogel thickness: 0.5 mm) at T= ~50 °C, which is above the transition temperature.

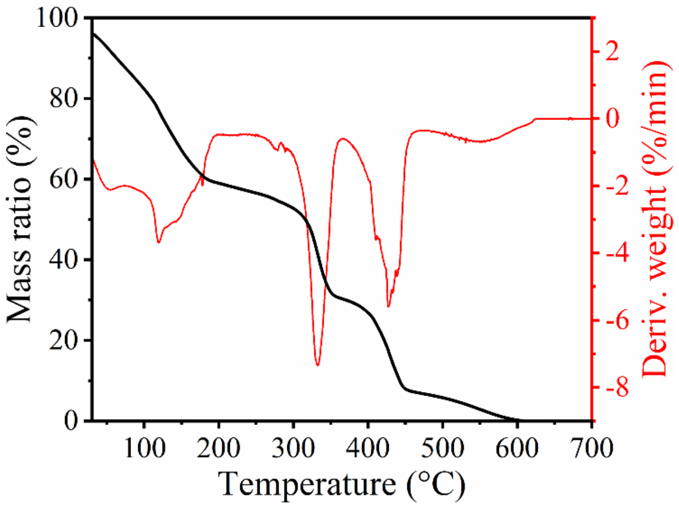

Supplementary Fig. 6: TGA of DMFM-4 hydrogel. TGA showed a 41% loss of weight that occurred below 185 °C and accelerated from 120 °C, which is associated with evaporation of water from the DMFM-4 hydrogel. As the temperature further increased up to 700 °C, the decomposition of the polymer moiety of DMFM-4 hydrogel could be observed. The water loss profile of DMFM-4 is similar to that of other hydrogel systems [1], showing the necessity for encapsulation.

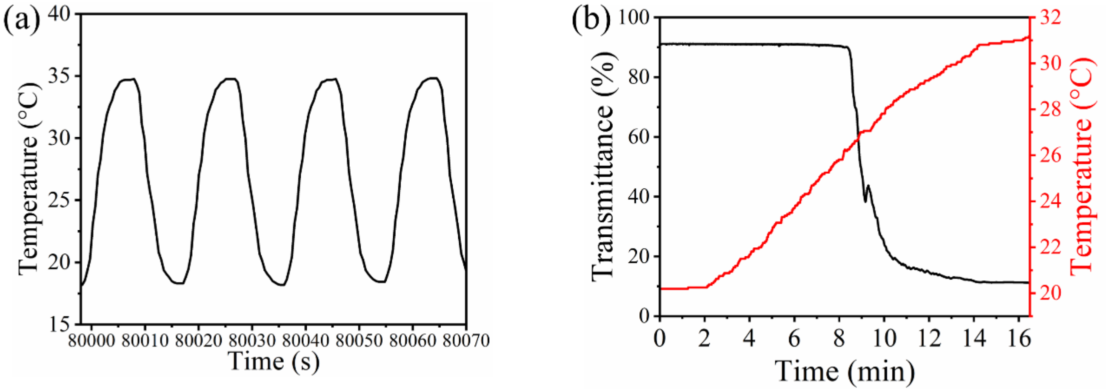

Supplementary Fig. 7: Stability and heating response of the DMFM-4 hydrogel device. (a) External temperature monitoring during the cyclic test of 10,000 heating/cooling cycles; (b) dynamic transmittance spectrum of DMFM-4 hydrogel device at 660 nm during slow heating process from 20 °C to 31 °C.

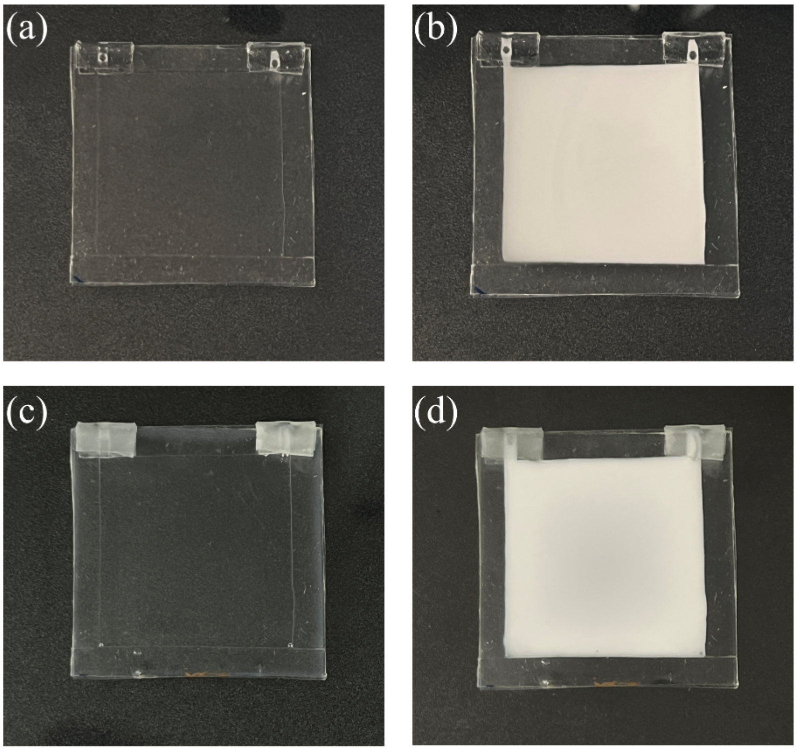

Supplementary Fig. 8: Optical images of DMFM-4 hydrogel device (4 cm * 4 cm *0.5 mm) before and after 10,000 heating-cooling cycles. (a) Images of DMFM-4 device under transparent state at 20 °C before 10,000 cycles; (b) under turbid state at 40 °C before 10,000 cycles; (c) under transparent state at 20 °C after 10,000 cycles; (d) under turbid state at 40 °C after 10,000 cycles.

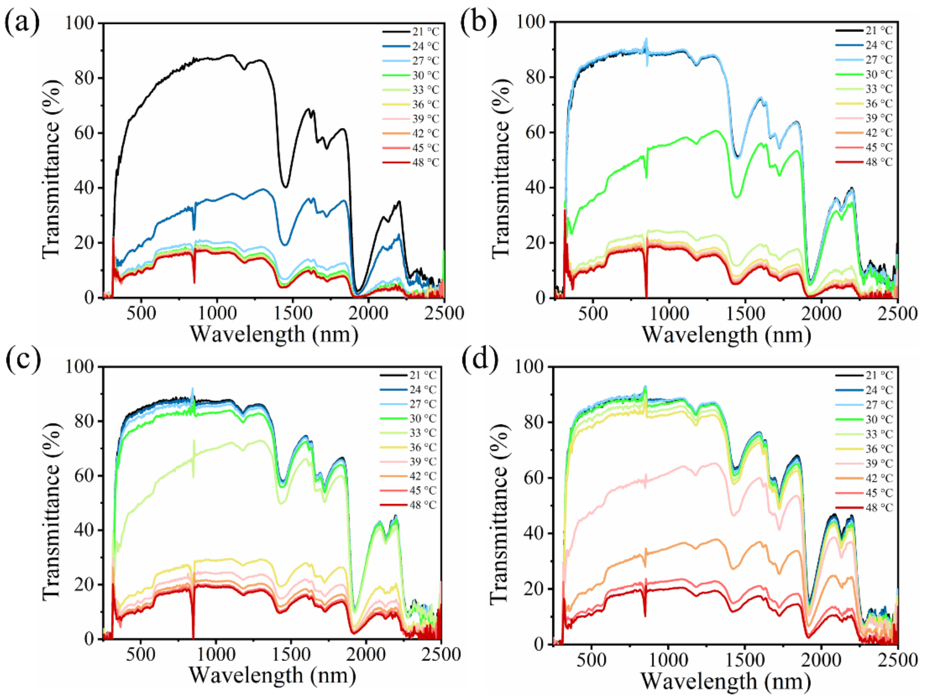

Supplementary Fig. 9: Thermochromic properties of DMFM hydrogels with different water contents. (a) Solar transmittance spectra (250 to 2500 nm) of the DMFM-5 hydrogel device with a layer thickness of 0.5 mm under temperature from 21 °C to 48 °C; (b) DMFM-3 hydrogel device; (c) DMFM-2 hydrogel device; (d) DMFM-1 hydrogel device.

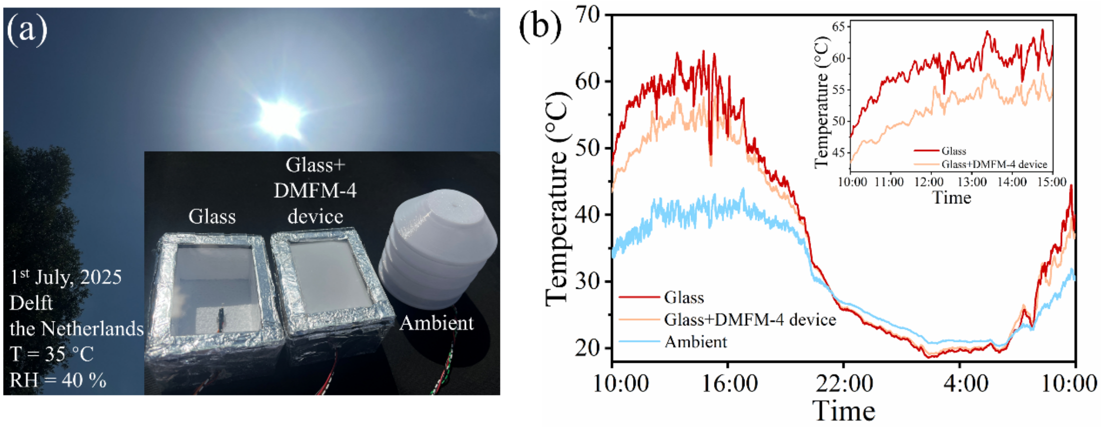

Supplementary Fig. 10: Outdoor temperature modulation by DMFM-4 hydrogel device. (a) Pictures of outdoor environment and simulation tests; (b) temperature profiles of temperature sensors in simulated houses (10 cm * 7.5 cm * 5 cm) with glass window or with DMFM-4 hydrogel device (8 cm * 5.5 cm * 0.5 mm) on the glass window, and in ambient environment exposed to outdoor solar radiation.

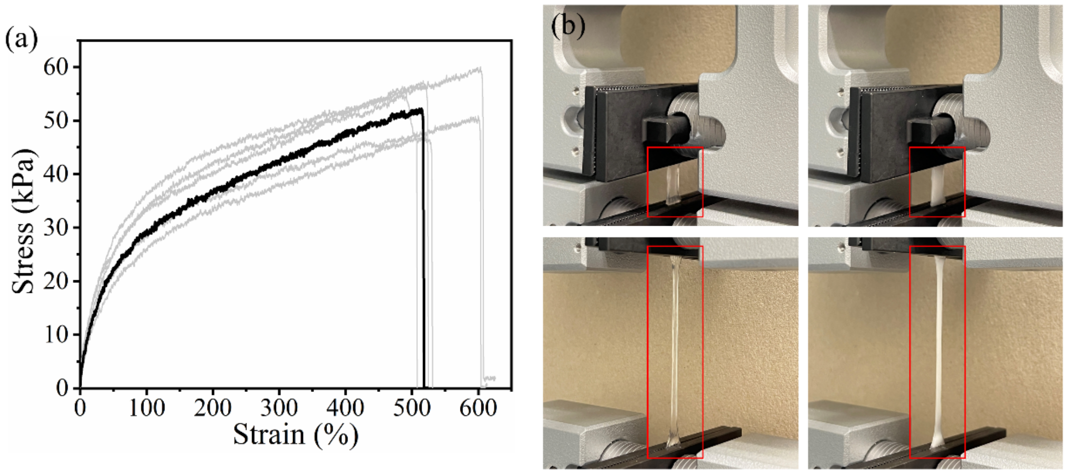

Supplementary Fig. 11: Tensile testing of DMFM-4 hydrogels. (a) Stress-strain curves from tensile tests on DMFM-4 hydrogels performed at a pulling rate of 10 mm min^{-1} (N = 6 DMFM-4 hydrogels); (b) pictures of pulling experiment of DMFM-4 hydrogel (corresponding to the black curve in panel a), in its relaxed (images on top) and stretched (500% strain) (images at the bottom) state, showing transparency at room temperature (images on the left) and turbidity when heated with an infrared lamp (images of the right).

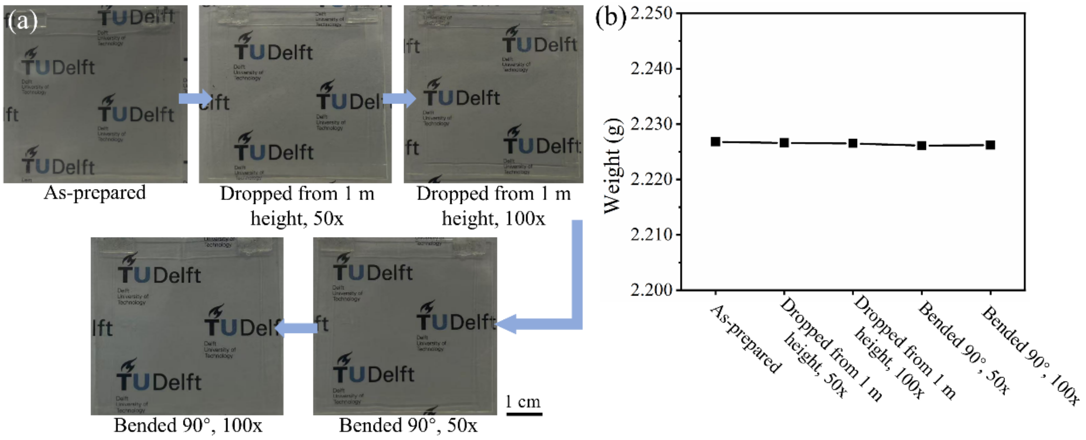

Supplementary Fig. 12: Anti-leakage testing. (a) Photographs of PET-encapsulated DMFM-4 hydrogel device (dimensions: 5 cm * 5 cm * 0.5 mm) in as-prepared state, after 50 and 100 drops from a height of 1 meter, after being bended 50 and 100 times to 90°; (b) the corresponding weight measurements of PET-encapsulated DMFM-4 hydrogel device under each condition.

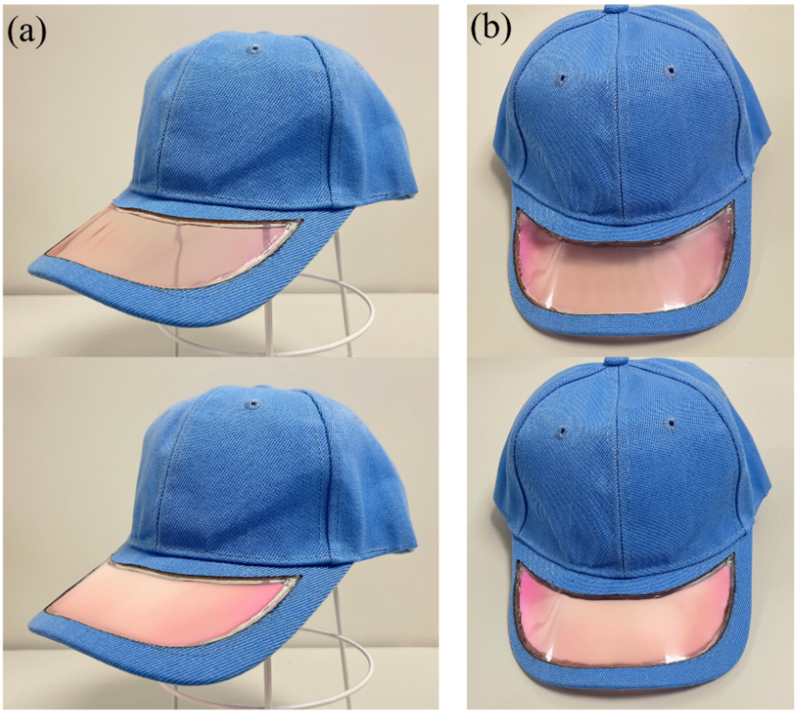

Supplementary Fig. 13: Thermochromic hat demonstration. (a) Side view and (b) top view of a flexible thermochromic hat device at transparent state (images above) and at turbid state (images at bottom, in which the columns of holder and shadow of hat were blocked by the turbid hydrogel).

Supplementary Table 1: The performances of current state-of-the-art thermochromic smart window systems.

ΔT_{solar}	T_{lum}	Transition temperature	Response time	Cycles	Solid State	Flexibility	Tunability	Materials	Fabrication process	Ref
7.50%	45.60%	68 °C	-	-	Yes	No	No	VO_2 film	Spin-coating and annealing	[2]
3.3-7.6%	47.5-48.9%	44.9-55.0 °C	-	-	Yes	No	Yes	Al-doped VO_2 film	Magnetron sputtering	[3]
1.0-5.0%	27.7-40.6%	34.4-50.7 °C	-	-	Yes	No	Yes	Mg-doped VO_2 film	Magnetron sputtering	[4]
3.3-7.4%	48.2-63.7%	26.0-57.0 °C	-	-	Yes	No	Yes	W-doped VO_2 film	Pulsed laser deposition	[5]
14.10%	60.40%	64.3 °C	-	-	Yes	Yes	No	Zr-doped VO_2 NPs in PU	Hydrothermal synthesis and curing	[6]
4.9-12.3%	48.6-58.4%	28.6-55.9 °C			Yes	Yes	Yes	Zr-W-doped VO_2 NPs in PU	Hydrothermal synthesis and curing	[6]
17.20%	53.00%	66.9 °C	-	-	Yes	Yes	No	Ti-doped VO_2 NPs in PU	Hydrothermal synthesis and curing	[7]
5.6-11.0%	45.3-54.2%	54-67 °C	-	-	Yes	Yes	Yes	Mg-doped VO_2 NPs in PU	Hydrothermal synthesis and curing	[8]
10.0-18.6%	63.3-82.5%	56.8-65.2 °C	-	-	Yes	Yes	Yes	$W_{18}O_{49}$-doped VO_2 NPs in PVP	Solvothermal synthesis and curing	[9]
37.70%	35.20%	65 °C	-	100	Yes	Yes	No	VO_2 NPs in PDMS	Mixing and curing	[10]
49.60%	85.80%	~32 °C	-	20	No	No	No	PNIPAM	Mixing and curing	[11]
34.70%	62.60%	35 °C	-	-	No	No	No	PNIPAM/VO_2	Mixing and curing	[12]
73.50%	88.00%	32.5 °C	17 s	200	Yes	Yes	No	PNIPAM microgel in Si/Al-gel	Mixing and curing	[13]
69.65%	87.37%	27.2°C	3 mins	100	No	No	No	PNIPAM/KCA/ Na_2SiO_3 suspension	Mixing and curing	[14]
68.10%	~90%	32.5 °C	-	100	No	No	No	PNIPAM liquid	Polymerization and freeze-drying	[15]
81.30%	87.20%	32 °C	-	1,000	No	No	No	PNIPAM-AEMA microgel	Mixing and curing	[16]
60.80%	89.20%	19.1 - 32.7 °C	9.6 s	10	No	No	Yes	PNIPAM GW solutions	Mixing and curing	[17]
47.50%	90.10%	10 - 44 °C	184 s	100	No	No	Yes	HPC/PAAc	Mixing	[18]
69.50%	84.40%	33.1 - 47.8 °C	20 s	50	Yes	No	Yes	P(AAm-co-AA)/NIPAM/AAm hydrogel	Mixing and curing	[19]
81.52%	90.82%	32 °C	-	100	Yes	No	No	PNIPAM/HPMC hydrogel	Mixing and curing	[20]
87.50%	71.20%	24.1 - 33.2 °C	40 s	100	Yes	No	Yes	HBPEC/PNIPAM hydrogel	Mixing and curing	[21]
61.36%	59.24%	32.9 °C	-	20	Yes	No	No	PNIPAm-acrylic/Ag NRs hybrid hydrogel	Mixing and curing	[22]
66.90%	51.20%	22 - 50.2 °C	30 s	100	Yes	No	Yes	HPMC/PDMAA hydrogel	Mixing and curing	[23]
-	-	25 - 45 °C	-	-	Yes	Yes	Yes	HPC/NaCl composite hydrogel	Mixing and curing	[24]
66.80%	87.00%	20 - 100 °C	3 mins	5,000	Yes	Yes	Yes	PU gel with ionic liquids	Mixing and curing	[25]
-	-	47 - 52.5 °C	-	-	Yes	Yes	Yes	P(DMAEMA-co-COU) hydrogel	Mixing and curing	[26]
-	76.40%	40 - 52 °C	28 s	500	Yes	Yes	Yes	P(DMAEMA-co-TSPM) hydrogel	Mixing and curing	[27]
**70.64%**	**85.67%**	**24 - 39 °C**	**3 s**	**10,000**	**Yes**	**Yes**	**Yes**	**P(DMAEMA-FMA) hydrogel**	**Mixing and curing**	**This work**

REFERENCES

[1] Bierbrauer K. L., Alasino R. V., Barclay F. E., et al. Biocompatible hydrogel for intra-articular implantation comprising cationic and anionic polymers of natural origin: In vivo evaluation in a rabbit model. *Polymers*, **2021**, 13(24): 4426.

[2] Zhou J., Gao Y., Zhang Z., et al. VO_2 thermochromic smart window for energy savings and generation. *Sci. Rep.*, **2013**, 3(1): 3029.

[3] Ji C., Wu Z., Wu X., et al. Al-doped VO_2 films as smart window coatings: reduced phase transition temperature and improved thermochromic performance. *Sol. Energy Mater. Sol. Cells*, **2018**, 176: 174-180.

[4] Panagopoulou M., Gagaoudakis E., Boukos N., et al. Thermochromic performance of Mg-doped VO_2 thin films on functional substrates for glazing applications. *Sol. Energy Mater. Sol. Cells*, **2016**, 157: 1004-1010.

[5] Bleu Y., Bourquard F., Barnier V., et al. Towards room temperature phase transition of W-doped VO_2 thin films deposited by pulsed laser deposition: thermochromic, surface, and structural analysis. *Materials*, **2023**, 16(1): 461.

[6] Shen N., Chen S., Chen Z., et al. The synthesis and performance of Zr-doped and W-Zr-codoped VO_2 nanoparticles and derived flexible foils. *J. Mater. Chem. A*, **2014**, 2(36): 15087-15093.

[7] Chen S., Dai L., Liu J., et al. The visible transmittance and solar modulation ability of VO_2 flexible foils simultaneously improved by Ti doping: an optimization and first principle study. *Phys. Chem. Chem. Phys.*, **2013**, 15(40): 17537-17543.

[8] Zhou J., Gao Y., Liu X., et al. Mg-doped VO_2 nanoparticles: hydrothermal synthesis, enhanced visible transmittance and decreased metal–insulator transition temperature. *Phys. Chem. Chem. Phys.*, **2013**, 15(20): 7505-7511.

[9] Qian J., Li B., Tian S., et al. Near-infrared-activated VO_2 based nanothermochromic smart windows by incorporation of photothermal $W_{18}O_{49}$ nanorods. *Appl. Surf. Sci.*, **2022**, 605: 154680.

[10] Ke Y., Yin Y., Zhang Q., et al. Adaptive thermochromic windows from active plasmonic elastomers. *Joule*, **2019**, 3(3): 858-871.

[11] Zhou Y., Cai Y., Hu X., et al. Temperature-responsive hydrogel with ultra-large solar modulation and high luminous transmission for “smart window” applications. *J. Mater. Chem. A*, **2014**, 2(33): 13550-13555.

[12] Zhou Y., Cai Y., Hu X., et al. VO_2/hydrogel hybrid nanothermochromic material with ultra-high solar modulation and luminous transmission. *J. Mater. Chem. A*, **2015**, 3(3): 1121-1126.

[13] Zhou Y., Layani M., Wang S., et al. Fully printed flexible smart hybrid hydrogels. *Adv. Funct. Mater.*, **2018**, 28(9): 1705365.

[14] Guo R., Shen Y., Chen Y., et al. KCA/Na_2SiO_3/PNIPAm hydrogel with highly robust and strong solar modulation capability for thermochromic smart window. *Chem. Eng. J.*, **2024**, 486: 150194.

[15] Zhou Y., Wang S., Peng J., et al. Liquid thermo-responsive smart window derived from hydrogel. *Joule*, **2020**, 4(11): 2458-2474.

[16] Li X. H., Liu C., Feng S. P., et al. Broadband light management with thermochromic hydrogel microparticles for smart windows. *Joule*, **2019**, 3(1): 290-302.

[17] Li G., Chen J., Yan Z., et al. Physical crosslinked hydrogel-derived smart windows: anti-freezing and fast thermal responsive performance. *Mater. Horiz.*, **2023**, 10(6): 2004-2012.

[18] Zhang L., Xia H., Xia F., et al. Energy-saving smart windows with HPC/PAA hybrid hydrogels as thermochromic materials. *ACS Appl. Energy Mater.*, **2021**, 4(9): 9783-9791.

[19] Wang W., Wang K., Cheng Y., et al. Bidirectional temperature-responsive thermochromic hydrogels with adjustable light transmission interval for smart windows. *Adv. Funct. Mater.*, **2024**: 2413102.

[20] Wang K., Chen G., Weng S., et al. Thermo-responsive poly (N-isopropylacrylamide)/hydroxypropylmethyl cellulose hydrogel with high luminous transmittance and solar modulation for smart windows. *ACS Appl. Mater. Interfaces*, **2023**, 15(3): 4385-4397.

[21] Sun M., Sun H., Wei R., et al. Energy-efficient smart window based on a thermochromic hydrogel with adjustable critical response temperature and high solar modulation ability. *Gels*, **2024**, 10(8): 494.

[22] Wei G., Yang D., Zhang T., et al. Thermal-responsive PNIPAm-acrylic/Ag NRs hybrid hydrogel with atmospheric window full-wavelength thermal management for smart windows. *Sol. Energy Mater. Sol. Cells*, **2020**, 206: 110336.

[23] Wang K., Liu S., Yu J., et al. Hofmeister effect-enhanced, nanoparticle-shielded, thermally stable hydrogels for anti-UV, fast-response, and all-day-modulated smart windows. *Adv. Mater.*, **2025**, 37(14): 2418372.

[24] Guo N., Liu S., Chen C., et al. Outdoor adaptive temperature control based on a thermochromic hydrogel by regulating solar heating. *Sol. Energy*, **2024**, 270: 112405.

[25] Lee H. Y., Cai Y., Velioglu S., et al. Thermochromic ionogel: a new class of stimuli responsive materials with super cyclic stability for solar modulation. *Chem. Mater.*, **2017**, 29(16): 6947-6955.

[26] Zhang W., Chen W., Lv J., et al. Multi-responsive P(DMAEMA-co-COU) hydrogel for temperature sensor and information encryption. *Eur. Polym. J.*, **2023**, 198: 112433.

[27] Kang S. K., Ho D. H., Lee C. H., et al. Actively operable thermoresponsive smart windows for reducing energy consumption. *ACS Appl. Mater. Interfaces*, **2020**, 12(30): 33838-33845.
